# From sympathetic overdrive phenotype to malignant transformation: 3PM-guided protection against health-to-disease transition and disease progression improving individual outcomes in primary and secondary care

**DOI:** 10.1007/s13167-026-00447-6

**Published:** 2026-04-29

**Authors:** Peter Kubatka, Andrea Kapinova, Bianka Bojkova, Vennila Joseph, Slavomir Hornak, Karol Kajo, Dietrich Büsselberg, Karel Smejkal, Vadim Goncharenko, Barbara Mrazova, Miroslava Fövényes, Rostyslav Bubnov, Walther Kuhn, Javier Conde-Aranda, Ivica Smokovski, Ivana Stetkarova, Olga Golubnitschaja

**Affiliations:** 1 Department of Natural Drugs, Faculty of Pharmacy, Masaryk University, 612 00 Brno, Czech Republic; 2 European Association for Predictive, Preventive and Personalized Medicine (EPMA), 1160 Brussels, Belgium; 3 Biomedical Centre Martin, Jessenius Faculty of Medicine, Comenius University in Bratislava, Mala Hora 4D, 03601 Martin, Slovakia; 4 Department of Animal Physiology, Institute of Biology and Ecology, Faculty of Science, Pavol Jozef Šafárik University in Košice, Košice, 040 01 Slovakia; 5 Department of Physiology and Biophysics, Weill Cornell Medicine in Qatar, Education City, Qatar Foundation, 24144 Doha, Qatar; 6 Small Animal Clinic, University of Veterinary Medicine and Pharmacy, 041 81 Kosice, Slovakia; 7 Department of Pathology, St. Elisabeth Oncology Institute, 812 50 Bratislava, Slovakia; 8 The Women’s Health Centre, Feofaniya Clinical Hospital of the State Management of Affairs of Ukraine, 21 Akademika Zabolotnoho St., Kyiv, Ukraine; 9 F. D. Roosevelt University Hospital, Banska Bystrica, Slovakia; 10 PREVITA Centrum pre prediktívnu, preventívnu a personalizovanú medicínu (PPPM), F. D. Roosevelt University Hospital, Banska Bystrica, Slovakia; 11 Department of Medical Imaging, Feofaniya Clinical Hospital of the State Management of Affairs of Ukraine, 21 Akademika Zabolotnoho St., Kyiv, Ukraine; 12 Zabolotny Institute of Microbiology and Virology, National Academy of Sciences of Ukraine, Kyiv, Ukraine; 13 Department of Gynecology and Obstetrics, Donau-Isar Klinikum Hospital, University Medical Campus Lower Bavaria, Deggendorf, Germany; 14 Molecular and Cellular Gastroenterology Group, Health Research Institute of Santiago de Compostela (IDIS), SERGAS, Travesia choupana s/n, Santiago de Compostela, Spain; 15 University Clinic of Endocrinology, Diabetes and Metabolic Disorders, Skopje, North Macedonia; 16 Faculty of Medical Sciences, University Goce Delcev, Stip, North Macedonia; 17 Department of Neurology, Charles University, 3rd Faculty of Medicine and University Hospital FNKV, Prague 10, Srobarova 50, 10000 Czech Republic; 18 Predictive, Preventive and Personalized (3P) Medicine, Department of Radiation Oncology, University Hospital Bonn, Rheinische Friedrich-Wilhelms-Universität Bonn, 53127 Bonn, Germany

**Keywords:** Predictive Preventive Personalized Medicine (PPPM / 3PM), Sympathoexcitation, Flammer syndrome, Endothelin-1 (ET-1), HIF, Ischemia-reperfusion, Chronic dehydration, Sterile inflammation, Xerostomia / Hyposalivation, Dry mouth syndrome, Burning mouth syndrome, Vulvar-vaginal dryness, Sicca syndrome, Sjögren syndrome, Chronic pelvic pain syndrome, Chronic prostatitis, Interstitial cystitis, Irritable bowel syndrome, Breast cancer, Colorectal carcinoma, Tumor microenvironment, Tumor hypoxia, Treatment resistance, Patient phenotyping and stratification

## Abstract

Chronic sympathoexcitation (sympathetic overdrive syndrome) is defined as the sustained dominance of sympathetic over parasympathetic tone, measurable at the organ, vascular, and neural levels, and differs fundamentally from brief, adaptive “fight-or-flight” responses. Persistent vasoconstrictor drive perturbs cellular metabolism and reshapes host–tumor interfaces. Although this state is well characterized in cardiometabolic medicine, its oncologic implications remain comparatively underexplored, despite converging evidence that adrenergic inputs modulate tumor and stromal behavior.

One of the main reasons for presenting this conceptual innovation study is that the sympathetic overdrive phenotype (SOP) carriers, by far, are not rare in the population. Robust statistics towards SOP incidence and prevalence are still missing, despite the urgency of plausible healthcare solutions. Currently, the best described SOP-relevant subpopulation are the Flammer Syndrome phenotype (FSP) carriers who, for instance, are highly prevalent in the academic career-making professional groups.

Certain genetic predispositions play a role, however, the key pathways involved into the health-to-disease transition are epigenetically regulated and, to a large extent, represent systemic modifiable phenotype-associated risk factors which are, therefore, a promising target for holistic proactive medical approaches with a high potential to save lives and financial resources.

**Graphical Abstract:**

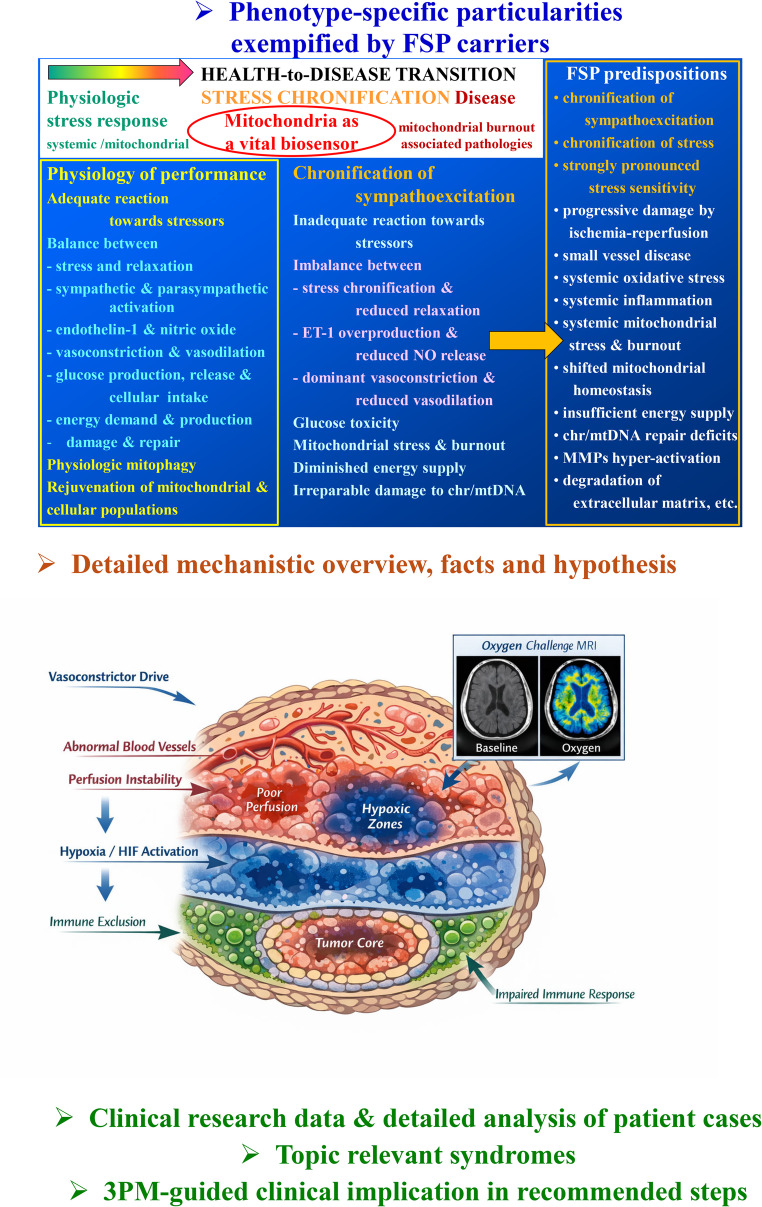

## Preamble

One of the main reasons for presenting this conceptual innovation study is that the sympathetic overdrive phenotype (SOP) carriers, by far, are not rare in the population. Robust statistics on SOP incidence and prevalence are still missing, despite the urgency of plausible healthcare solutions. Currently, the best described SOP-relevant subpopulation are the Flammer Syndrome phenotype (FSP) carriers who, for instance, are highly prevalent in the academic career-making professional groups. For example, amongst medical students, the FSP carriers may reach up to 70% [1].

**Fig. 1:**
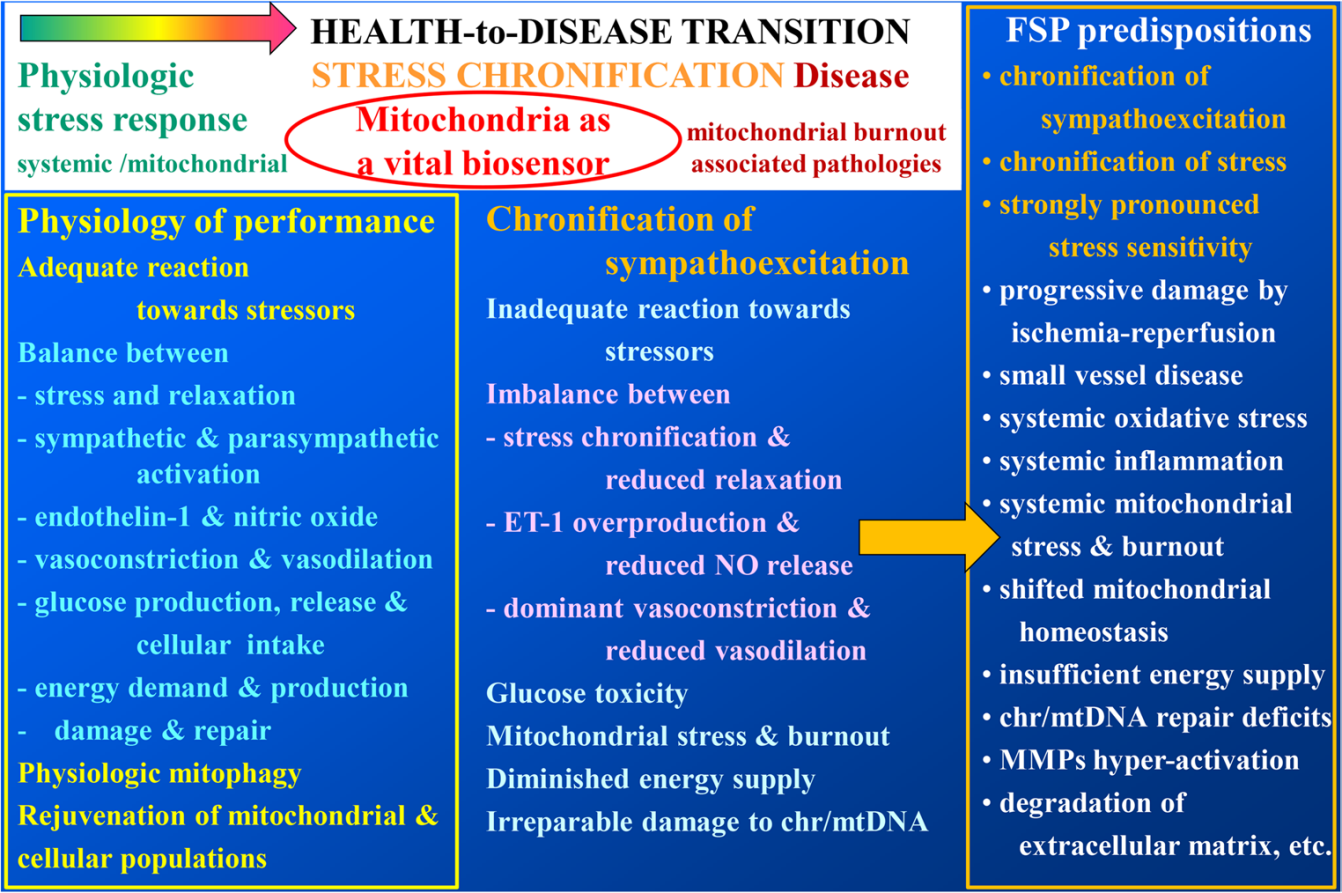
Sympathetic overdrive, stress chronification, and health-to-disease transition attributes characteristic of the FSP carriers; the figure is adapted from [2]

FSP carriers are usually success-oriented and highly stress-sensitive individuals; these phenotype-associated characteristics are essentially associated with significant health risks summarized in Fig. 1 [2], such as stress chronification, dominant vasoconstriction, chronic inflammation, MMPs hyperactivation, and extensive degradation of extracellular matrix, impaired wound healing, mitochondrial burnout, and insufficient energy supply for the repair machinery – all are the well-known attributes of malignant cell transformation and particularly aggressive cancer subtypes [3–6].

Certain genetic predispositions play a role; however, the key pathways involved in the health-to-disease transition are epigenetically regulated and, to a large extent, represent systemic modifiable phenotype-associated risk factors that are, therefore, a promising target for holistic proactive medical approaches with a high potential to save lives and financial resources.

This article presents a conceptual innovation that supports the paradigm shift from reactive to proactive medical services, using 3PM instruments to meet the needs of SOP carriers.

3PM-guided primary and secondary care deals with the following challenges:

- Phenotypic characterisation of the chronic sympathoexcitation.
- SOP characteristic mechanisms relevant for the malignant transformation.
- Disease-specific biomarker panels and multiparametric analysis for health risk assessment and predictive diagnostics applicable to the SOP carriers.
- SOP as the tool for population screening in primary care at the level of suboptimal health conditions (reversible damage to health), followed by the targeted disease prevention.
- Individualised patient profiles of the SOP carriers for the tailored treatment algorithms and digital health monitoring in primary and secondary care.

## Introduction

Chronic sympathoexcitation - defined as the sustained dominance of sympathetic over parasympathetic tone, measurable at the organ, vascular, and neural levels - differs fundamentally from brief, adaptive “fight-or-flight” responses [7]. Persistent vasoconstrictor drive perturbs cellular metabolism and reshapes host–tumor interfaces. Although this state is well characterized in cardiometabolic medicine, its oncologic implications remain comparatively underexplored, despite converging evidence that adrenergic inputs modulate tumor and stromal behavior [8, 9].

Physiological sympathetic spikes (e.g., cold pressor, orthostasis, mental stress) are transient and well captured by standardized provocations and direct neural recordings [7]. In contrast, the chronic phenotype features sustained vasoconstrictor outflow and catecholamine signaling that can be tracked longitudinally [10]. Microneurography provides beat-to-beat quantification of muscle sympathetic nerve activity (MSNA; bursts/min, bursts/100 heartbeats), making this distinction explicit and offering a mechanistic reference for clinical surrogates [11].

Several complementary readouts anchor the phenotype. Short-term heart rate variability (HRV) typically shows reduced high-frequency (HF) power and an increased LF/HF ratio, indicating vagal withdrawal and sympathetic predominance; these measures are feasible in clinic and wearable settings, with the caveat that LF and LF/HF require careful interpretation [12]. MSNA directly indexes vasoconstrictor outflow and correlates with higher peripheral resistance and cardiometabolic states relevant to cancer risk and survivorship [13]. At the molecular level, catecholamine/β-adrenergic signaling in tumor, endothelial, and immune compartments regulates proliferation, invasion, angiogenesis, and antitumor immunity [8]. Endothelin-1 (ET-1), a potent vasoconstrictor integrating autonomic and endothelial axes, is closely linked to hypoxia-inducible pathways within the tumor microenvironment (TME) [14].

These autonomic–vascular alterations matter for oncology because hypoxia and perfusion heterogeneity are central TME features that drive angiogenesis, immune exclusion, epithelial–mesenchymal transition (EMT), and treatment resistance [15]. Chronic sympathoexcitation can stabilize these conditions via sustained vasoconstriction (ET-1↑, MSNA↑), cyclic micro-ischemia–reperfusion, and amplified β-adrenergic cues across tumor and stroma [16], thereby connecting a measurable host state to canonical cancer hallmarks [17]. Preclinical evidence supports this link: repeated psychoemotional (immobilization) stress significantly worsened NMU-driven mammary tumorigenesis in vivo (higher incidence and burden; earlier onset) [18]. Moreover, a particular marker of chronic sympathoexcitation, such as ET-1, was highly expressed within colonic tumor tissue as compared to the adjacent normal mucosa [19], and ET-1 receptor signaling promoted EMT and chemoresistance in ovarian cancer xenograft mouse models [20].

Within the framework of Predictive, Preventive and Personalized Medicine (3PM/PPPM), chronic sympathoexcitation is reframed from a descriptive comorbidity to an operational biomarker axis: predict risk before therapy, prevent microvascular/metabolic deterioration, and personalize adjuncts in synchrony with tumor-directed regimens [21, 22].

Chronic sympathoexcitation constitutes a measurable and potentially modifiable host phenotype that predicts microvascular and metabolic vulnerability, supports preventive leverage against TME hypoxia and adrenergic signaling, and enables personalization of adjuncts alongside tumor-directed therapy. Positioned within 3PM, the progression from phenotyping to risk tiering, targeted action, and re-phenotyping is ready for prospective evaluation in oncology cohorts and window-of-opportunity trials [21].

### Study Aims and Hypotheses

#### Primary Aim

Validate a composite Sympathetic Overdrive Phenotype Score (5-min HRV, vitals, ET-1, symptoms ± MSNA) as a predictor of TME hypoxia/perfusion heterogeneity and early resistance; show ≤ 10-min, multicenter feasibility. 

H1: Higher baseline score → greater hypoxia/perfusion heterogeneity & early resistance [15, 23].

H2: Intake metrics are reproducible across sites per HRV standards [24].

#### Secondary Aim 1 **(Mechanistic)**

Link LF/HF, HF, ET-1, MSNA to mechanistic anchors.

H3: ET-1 and LF/HF associate with hypoxia volume and spatial immune exclusion [25, 26].

#### Secondary Aim 2 **(Preventive Responsiveness)**

Test short-cycle change in ET-1/HRV after preventive bundles (exercise, OSA care, stress/vagal-tone, BP/volume).

H4: Bundles reduce ET-1 and improve HF/LF-to-HF within one cycle [27, 28].

#### Secondary Aim 3 **(Personalization)**

Use cycle-to-cycle re-scoring to adapt adjuncts (β-blocker candidacy, ET-1-aware tactics, vagal-tone strategies).

H5: Adaptive personalization lowers hypoxia burden and autonomic-toxicity signals [29].

#### Exploratory Aim**(Outcomes & Value)**

 Estimate effect sizes for response durability, progression-free survival (PFS), tolerability, and cost-effectiveness; assess subgroup modifiers.

H6: Baseline score and early improvement predict longer PFS/better tolerance; phenotype-guided care is cost-effective [21, 23].

#### Sources of Research Data & Eligibility

 Data sources (PubMed, last search December 2025). Queries combined MeSH/keywords for: sympathetic/autonomic measures (HRV, BRS, MSNA), vascular markers (ET-1, capillaroscopy), TME biology (hypoxia, perfusion MRI, HIF, immune exclusion), and interventions (β-blockers, exercise, CPAP/OSA, HRV-biofeedback, tVNS), plus 3PM/PPPM terms. 

#### Inclusion criteria

- Original human/animal/translational data linking HRV/BRS/MSNA/ET-1/capillaroscopy to cancer/TME or treatment response.
- Controlled/trial designs testing mechanistic interventions (β-blockade, CPAP, exercise, HRV-biofeedback/tVNS, BP/volume optimization) with biomarker readouts (HRV, ET-1, perfusion/hypoxia MRI, immune spatial metrics).
- Studies operationalizing 3PM (risk stratification, tiered prevention, on-treatment personalization).
- High-quality methods/standards papers enabling these measures in oncology.

#### Exclusion criteria

- No cancer/TME relevance or no transferable mechanistic endpoint.
- Non-original research; incomplete methods or missing key acquisition/assay details (e.g., HRV without posture/artifact control; ET-1 without units/cut-offs).
- Interventions without autonomic/vascular biomarkers.
- Editorials/opinions/abstracts without full data; duplicate datasets (retain most complete).
- Non-oncologic models lacking hypoxia/perfusion or adrenergic/ET-1 applicability.

#### Scope notes

Prefer standardized protocols (HRV with posture/artifact control; single-platform ET-1 with defined ULN/CV; predefined imaging endpoints); treat MSNA as a research-grade validator.

## Host phenotyping: a pragmatic clinically relevant panel

This section operationalizes the diffuse “stress phenotype” into a clinic-ready, low-burden assessment that quantifies autonomic, vascular, and mito-metabolic status, yields a composite Sympathetic Overdrive Phenotype Score, and directly maps to 3PM actions - predictive cut-points, preventive bundles, and personalized adjuncts.

### Autonomic tests

#### **Short-term HRV (5 min)**

Acquire in a standardized, artifact-controlled posture; report HF power and LF/HF together with context (time of day, breathing, medications). A pattern of reduced HF with elevated LF/HF indicates vagal withdrawal and sympathetic predominance. Five-minute protocols are scalable and widely harmonized [24, 30].

#### **Resting HR/BP and baroreflex sensitivity (BRS)**

Resting tachycardia or hypertension adds independent prognostic information. BRS, an index of autonomic buffering, can be derived by sequence/spectral methods with growing standardization for clinical and ambulatory use [31].

#### **Optional MSNA (research subset) **

Peroneal microneurography provides beat-to-beat muscle sympathetic nerve activity (bursts·min⁻¹; bursts·100 beats⁻¹) as a mechanistic validator of vasoconstrictor outflow; best reserved for substudies and cross-site calibration [13].

### Vascular dysregulation

#### **Plasma endothelin-1 (ET-1)**

 ET-1 integrates autonomic and endothelial axes; elevated levels align with heightened vasoconstrictive tone, hypoxia biology, and tumor-promoting cues, making ET-1 a pragmatic proxy for vasoconstrictive load [32].

#### **Cold provocation capillaroscopy (where available)**

 Nailfold capillaroscopy non-invasively visualizes microvascular structure and reactivity; cold-challenge protocols reveal vasospastic tendencies relevant to perfusion instability [33].

### Mito-metabolic stress

#### **Lactate/pyruvate (L/P) ratio**

Increased lactate and an elevated L/P indicate redox/mitochondrial stress; interpretation must consider sampling conditions and comorbidities [34].

#### **Acyl-carnitine profile**

Circulating acyl-carnitines reflect β-oxidation flux and mitochondrial bottlenecks; profiles are increasingly deployed as systemic stress biomarkers across cardiometabolic and oncologic contexts [35].

#### **Fatigue indices**

 The 13-item FACIT-Fatigue is validated in oncology and is suitable for intake-level screening of energetic burden [36].

### Behavioral/constitutional traits

Stress sensitivity, perfectionism/rigor, and cold extremities frequently co-travel with primary vascular dysregulation (Flammer syndrome phenotype), signaling heightened vasoreactivity and mild ET-1 elevation - useful modifiers of risk interpretation [37].

### Output: The Sympathetic Overdrive Phenotype Score

Composite score from z-scaled components; weights calibrated prospectively.

- Autonomic: HF (−), LF/HF (+), resting HR (+), BRS (−); optional MSNA (+) in a research subset [13, 24, 38, 39].
- Vascular: ET-1 (+); capillaroscopy vasospasm index (+) [32].
- Mito-metabolic: L/P ratio (+), acyl-carnitines (+), FACIT-F (reverse) [34].

Report both the composite and raw components to retain transparency and enable targeted adjustments.

### 3PM mapping: from score to action

**Predictive (pre-therapy)**:

- Trial-calibrated cut-points (LF/HF↑ with HF↓, ET-1 > ULN, abnormal HR/BP or low BRS) flag microvascular fragility and higher risk of perfusion heterogeneity/hypoxia-driven resistance [12, 14, 24, 25, 30].

#### **Preventive (tiered bundles)**

 Match risk tier to mechanism-focused actions:

- Autonomic balance: structured exercise; screen/treat OSA (CPAP) as indicated; HRV-biofeedback or tVNS to raise vagal tone [40–43].
- Vascular tone: optimize BP/volume; consider ET-1-aware monitoring when ET-1 remains high [14].
- Adrenergic drive: when appropriate, β-blocker strategies under oversight, especially peri-operatively or in high-adrenergic states [44].
- Near-term markers - ET-1↓, HF↑, LF/HF↓ - guide rapid titration [45–47].

#### **Personalized (cycle-wise adaptation)**

Re-score each cycle and emphasize:

- ET-1/vasoconstriction → microvasculature-protective tactics and endothelial hygiene [14].
- β-adrenergic tone → β-blocker candidacy + stress-axis control (monitor HR/BP/HRV) [29].
- Vagal withdrawal → vagal-tone strategies (HRV-biofeedback, tVNS) [42, 43].

### Practicalities and harmonization

- Acquisition standards: HRV per task-force guidance (posture, artifact control, breathing); document meds/stimulants; specify BRS method [24, 30].
- Assay consistency: Single ET-1 platform across centers with predefined ULN and analytic CV [32].
- Substudy calibration: Include MSNA at sentinel sites to validate autonomic/ET-1 surrogates against direct neural outflow [13].

This phenotyping block is intentionally clinic-pragmatic: each readout maps to mechanisms that govern tumor perfusion and hypoxia, enabling an actionable 3PM loop - predict → prevent → personalize → re-score [15, 21]. The clinic-ready intake generates a composite Sympathetic Overdrive Phenotype Score (5-min HRV, vitals, ET-1, symptom screen ± MSNA) in ≤ 10 min. We will evaluate, prospectively and across centers, whether this score predicts TME hypoxia/perfusion heterogeneity and early treatment resistance.

## Mechanistic axes linking the phenotype to malignant transformation and cancer progression

### Microvasculature, hypoxia, and HIF-signaling

Stimulation of angiogenesis is one of the hallmarks of cancer cells [48], as a growing tumor requires an increased supply of oxygen and nutrients. However, tumor microvasculature is often poorly formed; the blood vessels are crooked and leaky, leading to uneven perfusion and fluctuating oxygen levels. This results in patchy hypoxia, where some regions of the tumor cycle between ischemia and reperfusion [49–51]. Hypoxia induces changes in hypoxia-inducible factor (HIF) signaling, increases metastasis, and supports resistance to chemotherapy and radiotherapy [52]. Additionally, hypoxia is associated with immune cell recruitment, and an increase in inflammatory mediators like cytokines and reactive oxygen species (ROS), which can lead to chronic inflammation [53, 54]. Abnormal tumor microvasculature, hypoxia, and inflammation promote an altered tumor microenvironment (TME) also via extracellular matrix remodelling (ECM), thus contributing to a more aggressive tumor phenotype [55, 56].

HIF-signaling is a crucial pathway that helps cells adapt to hypoxia by promoting survival, angiogenesis, and metabolic switch [57]. In cancer, however, this adaptive response becomes dysregulated and supports tumor growth [58]. HIF refers to a family of transcription factor complexes (HIF-1, HIF-2, and HIF-3), among them, HIF-1 acts as a master regulator of processes critical to cancer progression and survival [59].

Major HIF-1 targets regulating tumor angiogenesis include VEGF, a central mediator of angiogenesis, and the vasoconstrictor peptide ET-1. HIF-1 and ET-1 form a positive feedback loop that favours tumor angiogenesis and growth. HIF-1 directly promotes ET-1 expression by binding to its promoter. In turn, ET-1 enhances HIF-1 activity and stability, which creates a reinforcing cycle leading to further expression of HIF-1-regulated genes, including VEGF [32, 60]. However, VEGF may also be upregulated via HIF-independent mechanisms [61, 62].

Hypoxia is regarded as a major factor in a complex network of immune evasion strategies in tumors [63]. VEGF, ET-1, and another HIF target, CXCL12 (SDF-1), contribute to immune evasion by promoting T-cell exclusion from the tumor nests [64–66]. In addition, VEGF and CXCL12 recruit regulatory T cells (Tregs) and myeloid-derived suppressor cells (MDSCs), which impair T-cell functioning [67, 68]. Immune evasion is also fueled by a metabolic shift in TME towards aerobic glycolysis orchestrated by HIF target genes, leading to nutrient deprivation of infiltrating T-cells and NK cells and metabolic acidification, which promotes a pro-tumor M2 phenotype and suppresses T-cell activation [69, 70].

Patchy hypoxia in tumors also favours partial or full EMT largely through HIF-1 stabilisation [60, 71]. HIF-1 affects EMT by induction of key EMT-inducing transcription factors (e.g., Snail, Twist, ZEB), miRNA regulation, and epigenetic mechanisms [72, 73]. EMT drives metastasis, stemness, immune evasion, and drug resistance through complex crosstalk networks (e.g. PI3K/Akt/mTOR and Ras/MAPK), which in turn, reinforce EMT, creating a positive feedback loop [74–76].

Tumor microvascular dysfunction triggers cycles of ischemia/reperfusion that results in mitochondrial ROS surge [51, 77]. ROS bursts may drive EMT through HIF-1-independent mechanisms [78, 79], while also promoting DNA damage, genomic instability, and inflammatory pathways (e.g., NF-κB) that further stabilise HIF, overall promoting a more aggressive, hostile and treatment-resistant TME [51, 80]. Notably, the chemoresistance induced by the TME can be reversed by inhibiting HIF-1α in the context of colorectal cancer [81].

### MRI options and tissue profiling for detection of early-warning signals in high-score patients

#### **MRI hypoxia/perfusion anchors (with delta-readouts)**

Tumor hypoxia can be mapped non-invasively with oxygen-challenge MRI. Oxygen-enhanced MRI (OE-MRI) quantifies dissolved molecular O₂ and yields hypoxic-volume (HV) metrics that change early during therapy; paired work in head-and-neck cancer demonstrates measurable HV at baseline and significant early within-treatment reductions that track response, supporting OE-MRI as a biologically specific hypoxia readout [82]. Blood-oxygen-level–dependent (BOLD) and tissue-oxygen–level–dependent (TOLD) MRI complement OE-MRI by capturing oxygenation-linked signal changes during standardized O₂ challenges, while dynamic contrast-enhanced MRI (DCE-MRI) provides quantitative perfusion/permeability parameters (e.g., K^trans^, v_e_) that reflect macro- /microvascular function and treatment-induced vascular normalization. Collectively, these sequences enable “delta” assessment (weeks 2–4 in many protocols) of early trajectories - HV↓ and perfusion gain - before anatomic response is visible [83].

#### **Tissue and liquid profiling (hypoxia/immune axes)**

 To mechanistically anchor imaging, tissue panels sample canonical HIF-responsive targets and metabolic rewiring markers—CAIX (membranous hypoxia marker), GLUT1 (glucose transport), and LDHA (glycolysis)—together with angiogenic effectors (e.g., VEGF) and the CXCL12/CXCR4 chemokine axis that promotes T-cell exclusion and stromal recruitment. These are complemented by adenosine-pathway enzymes CD39/CD73, which generate immunosuppressive adenosine and correlate with poor immune infiltration; taken together, the panel links hypoxia and perfusion instability to immune evasion and EMT-biased biology [84].

**Fig. 2:**
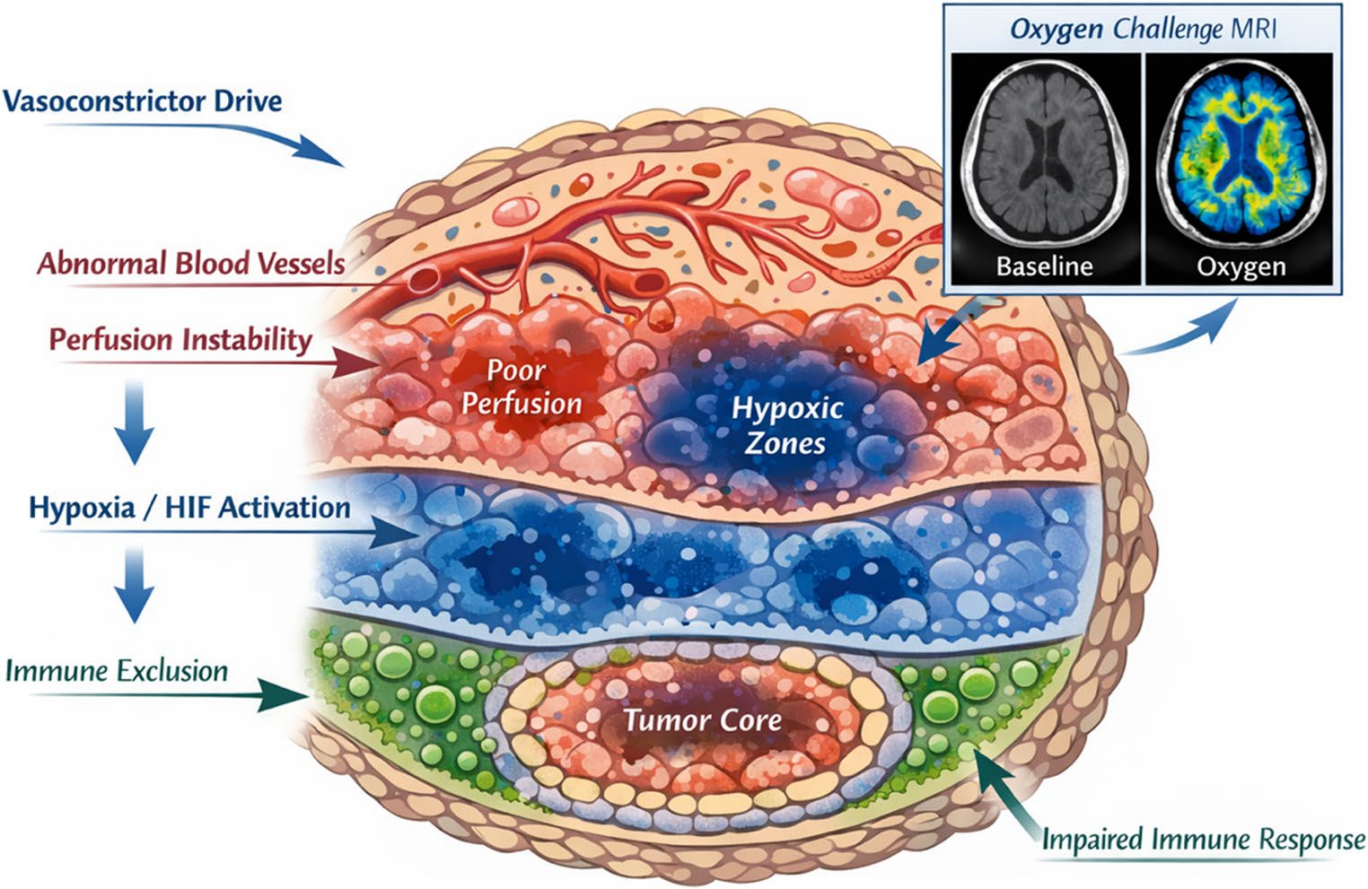
Mechanistic anchoring of host autonomic–vascular phenotype in the tumor microenvironment (TME)

The phenotype is not just upstream; it is inscribed into the tumor landscape. From vasoconstriction to hypoxia to immune exclusion, the host’s systemic state shapes the microenvironment’s resistance architecture (Fig. 2). This scheme illustrates the layered interplay between systemic host phenotypes and the intratumoral microenvironment. The top layer represents perfusion heterogeneity, highlighting abnormal vasculature and unstable flow driven by vasoconstrictor tone. The middle layer depicts patchy hypoxia and HIF pathway activation, mechanistically linked to perfusion deficits. An inset MRI (oxygen challenge) visualizes functional imaging validation of hypoxic zones. The bottom layer shows immune exclusion, where cytotoxic T cells are barred from tumor nests by stromal and vascular barriers, linking impaired immune access to upstream vascular dysfunction. Arrows on the margins trace a causal axis from host physiology to TME resistance, supporting a 3PM rationale for integrative monitoring and intervention.

### Modification of ET-1 signaling for microvasculature protection

ET-1 upregulation promotes vasoconstriction, inflammation, and endothelial dysfunction, which reinforce abnormal microvasculature within tumors [85, 86]. Therapeutic strategies aim to blunt ET-1 signaling through reduced ligand production, endothelin receptor antagonism, or improved nitric oxide balance. Preclinical and clinical studies suggest these interventions can stabilise microvascular function, enhance tissue perfusion and improve drug delivery, thereby overcoming drug resistance and boosting treatment efficacy [32, 87, 88].

Preventive intent is explicit: microvasculature-protective tactics should blunt ET-1–driven vasoconstriction and suppress the impact of cyclic ischemia–reperfusion with its ROS bursts that entrench HIF signaling and resistance. Beyond pharmacologic ET-1/NO modulation, it is essential to implement “endothelial hygiene” - BP/volume optimization, OSA management/CPAP, graded aerobic exercise, and avoidance of strong thermal/cold provocation—to stabilize perfusion before resistance consolidates [14, 45, 89, 90].

### Personalized co-interventions for cancer therapy

To optimise therapeutic outcome in high-score patients, the co-intervention should be customised to target the dominant pathway identified in the TME.

In patients with upregulated ET-1 and associated vasoconstriction, selective endothelin receptor antagonists (ERAs) or dual ERAs can be employed to block ETA receptors, which are often overexpressed in cancer cells and surrounding stroma [91, 92]. This inhibits ET-1-mediated signaling pathways involved in cancer proliferation and progression; therefore, even if ERAs have not been proven clinically to treat established cancers [93], they may be considered as a preventive measure.

Targeted mitochondria-specific therapies (mitocans) are convenient when mitochondrial ROS signaling is the primary driver. Cancer cells often exhibit higher basal levels of ROS than healthy cells, which makes them vulnerable to agents that further increase oxidative stress beyond the lethal threshold or disrupt mitochondrial membrane potential [94, 95]. Mitocans induce apoptosis by destabilising mitochondria and releasing pro-apoptotic factors, which can help overcome the drug resistance mechanisms [96, 97].

When neuroinflammation is the predominant mechanism, systemic interventions (e.g., beta-adrenergic receptor (β-AR) antagonists or anti-inflammatory agents) can disrupt the self-reinforcing circuit between the nervous and immune system that fuels cancer progression [16, 98]. Chronic sympathoexcitation increases norepinephrine and epinephrine, which bind to β-ARs (particularly β2-ARs) on immune and tumor cells. This leads to anti-tumor immune suppression, angiogenesis promotion, and increased release of inflammatory cytokines [99]. Non-selective beta-blockers (e.g., propranolol) can attenuate these effects, restore T-cell function and infiltration, and enhance the efficacy of chemotherapy and radiotherapy [100], adding to their cardioprotective effect in high-risk/cancer patients.

Personalization follows the Sympathetic Overdrive Phenotype Score: ET-1↑/capillaroscopy vasospasm **→** endothelin-aware tactics; β-adrenergic tone/HRV shift **→** β-blockade + stress-axis control; mito-ROS signatures **→** mitocans; neuroinflammatory skew **→** anti-inflammatory and vagal-tone–restoring approaches. Confirmation endpoints are prespecified: ET-1↓ with OE/BOLD-MRI perfusion gain; β-axis control → HF-HRV↑/LF: HF↓ with NE/metanephrine↓ and greater intratumoral CD8⁺ proximity; mito-ROS control → lower 4-HNE/mtDNA injury with EMT/HIF attenuation [88–103].

Harmonized intake uses artifact-controlled 5-min HRV with standardized posture/breathing, single-platform plasma ET-1 with predefined ULN/CV, and brief vitals/fatigue; mechanistic anchors include hypoxia/perfusion MRI, and tissue/immune panels are shown in Table 1.

**Table 1:** Intake panel and mechanistic anchors

Component	What to collect (standard)	Rationale for inclusion	References
5-min HRV	Artifact-controlled HF power, LF/HF; standardized posture; paced/documented breathing; report SDNN as available	Valid, consensus HRV acquisition and interpretation; autonomic (vagal/sympathetic) balance proxy	HRV Task-Force standard [23]; prognostic HRV in oncology [24]
Resting vitals & BRS	Resting HR/BP; baroreflex method specified	Captures basal sympathetic tone and buffering	HRV Task-Force (methods/BRS) [23]; peri-treatment BP targets [30]
Plasma ET-1 (single platform)	Same assay/site; predefined ULN and CV	ET-1 marks vasoconstrictive/microvascular dysfunction; links to cancer biology	Endothelin biology in cancer [32]; ET-1 dysregulation/microvascular dysfunction [25]
Brief Fatigue Scale	Short patient-reported fatigue	Captures the host burden affecting the autonomic tone	Brief patient-reported fatigue scale [36]
Optional nailfold capillaroscopy	Vasospasm index (where available)	Non-invasive microvascular readout complementing ET-1	Methods used for capillaroscopy [33]
OE-/BOLD-/TOLD-MRI	Hypoxic volume (OE/TOLD), oxygenation signal (BOLD), early “delta” response (weeks 2–4)	Early, non-invasive hypoxia mapping to anchor host phenotype	DCE/OE/BOLD usage in therapy evaluation [83, 84]; vessel-normalization framework [49, 90]
DCE-MRI perfusion	K^trans^, v_e_ (site standard)	Perfusion/permeability and early vascular-response biomarker	DCE-MRI biomarkers [103]
Tissue hypoxia & immune panels	CAIX, GLUT1, LDHA, HIF-sets; CXCL12/CXCR4; adenosine axis (CD39/CD73)	Links hypoxia/perfusion to immune exclusion and resistance pathways	Hypoxia/TME overview [17]; SNS–TME crosstalk [16]

## Up-to-date evidence in oncology – colorectal and breast cancer studies

### Core autonomic signal in CRC and BC: vagal withdrawal dominates

Chronic sympathoexcitation in cancer is most consistently seen as vagal withdrawal (low HF/RMSSD), with less uniform evidence for frank sympathetic overactivation; this mirrors broader findings in cardiometabolic disease and chronic conditions [104–113]. In oncology, data remain sparse and heterogeneous, yet autonomic imbalance appears linked to tumor biology and outcomes [114, 115]. Across CRC and BC, many cohorts show lower HRV (HF, RMSSD); sympathetic markers (catecholamines/LF metrics) vary by stage and treatment, so the more robust signal is reduced parasympathetic tone [116, 117].

### What do existing studies already capture, and what is still missing

Existing CRC/BC data already populate a Sympathetic Overdrive Phenotype Score (short-term HRV depression, subsets with resting tachycardia/hypertension, signals consistent with ET-1 activity). What’s missing are standardized 5-minute HRV (posture/breathing/artifact control), serial plasma ET-1 on a single platform (ULN/CV predefined), structured symptom/fatigue capture, and longitudinal linkage to mechanistic anchors (hypoxia/perfusion MRI, HIF targets, spatial immune metrics). Without these elements, studies remain cross-sectional and underpowered for prediction [30, 118].

HRV, primarily a noninvasive index of cardiac vagal tone, predicts adverse outcomes in CV and renal disease [119–123]. In cancer, higher HRV generally associates with better survival [23–127], plausibly via anti-inflammatory and immune-regulatory effects of the vagus [116]. Cancer therapy itself perturbs autonomic function and CV risk [128–130]; patients with cancer show lower HRV than controls, and advanced disease shows lower HRV than early stage [126, 127, 131].

### Colorectal cancer evidence and implications for prospective designs

In non-metastatic, surgery-treated CRC, large cohorts report no survival association for time-domain HRV (SDNN/RMSSD): McGovern et al. (*n* = 439) and Strous et al. (*n* = 428) both negative, despite prevalent low vagal tone; thresholds and medians differ across studies but remain in contemporary ranges [125, 127, 132–134]. HRV prognostic signals are more often reported in advanced/metastatic settings [132, 134–136]. Notably, Gidron et al. showed vagal activity moderates prognosis: low HRV allowed stage to predict higher follow-up tumor markers (CEA/PSA), whereas high HRV buffered this effect [136]. **Prospective CRC need**: Acquire a uniform intake battery (5-min HRV, resting HR/BP, ET-1, fatigue), repeat HRV/ET-1 on treatment, and co-register with perfusion/hypoxia MRI and spatial immunity to test “baseline + early change → resistance/tolerance.”

### Breast cancer evidence and translation to intake with monitoring

BC cohorts similarly show reduced parasympathetic activity and, in some studies, higher sympathetic modulation; HRV may flag patients who benefit from autonomic-targeted supports (psychotherapy, biofeedback, β-blockers) [137]. Lower HRV correlates with fatigue and inflammatory markers (IL-6, CRP) in early BC [138]; HRV also relates to tumor markers in small prospective work [139]. In metastatic BC, higher HF-HRV associates with longer survival [140]. A systematic review (12 studies) supports HRV as a complementary, noninvasive tool for autonomic dysfunction and outcome assessment, though predictive value remains incompletely defined [141]. Early BC translation: The same intake panel can be deployed pre-therapy to identify “microvascular-fragile” patients, defined by depressed HF-HRV and ET-1 above the laboratory ULN, then validated against OE/BOLD-MRI hypoxia and HIF-responsive targets. Cycle-wise re-scoring of HRV and ET-1 enables adaptive supportive care throughout treatment.

#### **Limitations and next steps**

Much of the current evidence comes from small, cross-sectional studies that are insufficiently adjusted for factors such as stage, treatment changes, or mechanistic mediators (e.g., cytokines, oxidative stress). HRV does not capture organ-specific vagal signaling; therefore, vagal-enhancing approaches (biofeedback, exercise, pharmacologic agents, vagal stimulators) require rigorous oncology trials [132, 142–148]. Moreover, vagal signaling may be context-dependent, with acetylcholine potentially promoting tumorigenesis in some settings, including CRC, necessitating tumor-type–specific evaluation [116, 149].

### Mechanistic pathways (β-adrenergic, ET-1–HIF/hypoxia) and the 3PM roadmap

#### **Catecholamines/β-adrenergic signaling**

β-AR pathways—particularly β2-AR—shape CRC/BC progression; The expression of these receptors has been linked to chemoresistance and prognosis, and perioperative β-blockade plus COX-2 inhibition favorably modulates metastasis, immune, and EMT biomarkers [150–155]. Observational data in BC associate β-blocker exposure with improved outcomes, but results are inconsistent and warrant controlled trials; circulating catecholamines and receptor profiles vary by subtype and stage [156–158].

#### **ET-1 and hypoxia-inducible pathways**

Hypoxia drives malignant progression and therapeutic resistance across CRC/BC [159–167]. ET-1 is overexpressed and stabilizes HIF-1α, augments VEGF, and activates PI3K/AKT/MAPK signaling, thereby promoting EMT and resistance [32–169]. Early trials of ET-receptor antagonists confirm safety but limited monotherapy efficacy, supporting combination strategies and predictive biomarkers [32, 168, 170, 171]. Systemically, low HRV and high ET-1 bias toward a hypoxia-dominated TME, linking host autonomic phenotype to resistance biology [32, 124, 172, 173]. Mitigation strategies include vagal-tone enhancement (biofeedback, exercise, stress management) and ET-axis targeting in ET-1↑/HRV↓ patients [174, 175].

#### **Phenotype–mechanism concordance**

 An ET-1–positive, vagal-low profile aligns with patchy hypoxia, HIF activation, and immune exclusion; embedding ET-1 and HRV in intake and monitoring provides a testable upstream host signal in CRC/BC

#### **3PM reinterpretation and prospective testing**

 Within a 3PM framework, baseline autonomic/ET-1 profiles serve as predictive selectors; predefine trajectory triggers (e.g., ≥ 20% rise in LF/HF or ET-1 within one cycle), implement tiered prevention (exercise, OSA/CPAP, BP/volume optimization, stress/vagal strategies; β-blockade where appropriate), and document personalization via improvements in perfusion/hypoxia imaging and tolerability (fewer DLTs, preserved dose intensity) [22]. CRC/BC already contains the requisite signals; the critical need is for harmonized, longitudinal, mechanism-anchored datasets to test whether phenotype-guided care improves tumor control and treatment tolerance [118].

## Diagnostics & trial-ready endpoints

We propose a multimodal diagnostic panel to quantify sympathetic excitability and vascular stress in cancer patients. The base panel comprises readily obtainable measures, 5-minute short-term HRV, resting HR/BP, plasma ET-1, and a brief fatigue instrument (e.g., FACIT-Fatigue), which were selected for feasibility and prognostic value. Lower short-term HRV (low HF, high LF/HF) associates with adverse outcomes [176]. For instance, in glioblastoma (*n* = 88), lower 5-minute HRV during initial supine rest predicted shorter survival (13.2 vs. 20.2 months; *p*<.05) [177]. In pancreatic cancer (*n* = 41), reduced SDNN independently predicted mortality (9 vs. 15 months; adjusted HR ≈ 2.30; *p* = .034) [178]. Resting tachycardia adds risk information: among 548 treatment-naïve patients, each 5-bpm increase in resting HR raised all-cause mortality by ~ 10% (adjusted HR 1.10 per 5 bpm; *p*<.001) [179].

ET-1 complements autonomic indices as a systemic marker of vasoconstrictive load linked to hypoxia and invasion; higher tumor/endothelial ET-1 aligns with aggressive phenotypes [180, 181], whereas suppression reduces aggressiveness in model systems [182]. Serial plasma ET-1 (and/or big ET-1), interpreted alongside vitals, therefore supports identification and monitoring of patients vulnerable to microvascular dysfunction. Patient-reported fatigue integrates symptom biology with low burden: FACIT-Fatigue (0–52; <34 denotes significant fatigue) provides outcome-relevant signal [183]. Because sympathoexcitation and sleep disruption contribute to cancer-related fatigue [184, 185], combining FACIT-Fatigue with HRV strengthens early detection of at-risk patients.

### Advanced Measures of Vascular and Neurofunctionality

For mechanistic resolution, flow-mediated dilation (FMD) indexes macrovascular endothelial function; lower FMD reflects dysfunction [186] and is commonly observed after cancer therapy [187]. Exercise can improve FMD [188], positioning it as both a risk indicator and intervention endpoint. To capture microvascular behavior, nailfold capillaroscopy with cold provocation visualizes vasospastic reactivity; exaggerated constriction and slow reperfusion (Flammer-like response) suggest systemic dysregulation [189], consistent with the sensitivity of tumors to microvascular flow instability [49].

A neuroendocrine lens can be added with catecholamines, which are elevated in some cancers (e.g., oral cancer) [190], though variability limits their routine use. Resting-state fMRI reveals treatment-related network alterations, particularly within default-mode and fronto-parietal systems after chemotherapy [191], and may serve as a biomarker of neurotoxicity risk.

### Digital Phenotyping

Wearables devices extend the panel longitudinally by continuously tracking HR, HRV, activity, and sleep with low patient burden. In lung cancer, 24-hour wearable HRV (LF/HF) differentiated fatigue severity with 73–88% accuracy [192]. Wearable sleep estimates align well with diary-based measures in oncology cohorts [193]. These data streams can function as trial-ready endpoints and practical triggers (e.g., rising LF/HF or decreasing sleep time) for timely clinical review and reinforcement of preventive bundles, translating point-in-time autonomic assessment into continuous, patient-centric monitoring.

### Endpoint Framework: Predictive, Preventive, Personalized

#### Predictive

 Baseline phenotyping will stratify risk using explicit thresholds and early trajectories. Patients with LF/HF > 2.5 [194], SDNN decline > ~ 2.3% over the first 6 weeks with larger HF drops (> 15%) [195], or persistent ET-1 ≳ 5 pg/mL [196, 197], comprise higher-risk strata for chemotherapy complications and hypoxia-prone TME biology. Operationally, individuals showing SDNN ↓ >2–3% over 6 weeks or ET-1 ≥ 5 pg/mL will be auto-flagged for vasculoprotective/preventive interventions, enabling prospectively defined, trial-ready triggers for action. 

#### **Preventive**

 Abnormal baseline or early trajectory signals will trigger targeted prehabilitation, prioritized based on the underlying mechanisms. Core components include supervised exercise to improve autonomic balance (HRV) and endothelial function (FMD) [188, 198], and CPAP to blunt nocturnal sympathetic surges in patients with OSA [28]. In a 16-week prospective trial, moderate aerobic training increased HRV by 11% during and 16% after treatment versus declining controls [27]; aerobic exercise can also reduce ET-1 by approx 20% over approximately. 3 months [199]. These modalities provide short-cycle titration endpoints (HF↑, LF/HF↓, ET-1↓), enabling cycle-to-cycle adjustment of the bundle.

#### **Personalized**

When available, integrate early “delta” hypoxia/perfusion readouts (e.g., weeks 2–4) with HRV/ET-1 trajectory to adapt adjunct strategy in real time. Imaging evidence of persistent hypoxia or absent perfusion gain supports escalation of microvasculature-protective and/or hypoxia-directed components, whereas an early favorable delta supports de-escalation or maintenance to minimize unnecessary adjunct burden [118]. Technical acquisition and interpretation of OE/BOLD/TOLD/DCE-MRI and hypoxia-volume/perfusion metrics are specified in Sect. 3.2.

#### **Tolerability / DLT endpoints**

Dose-limiting toxicities (DLTs) will be defined per CTCAE v5.0 (grade ≥ 3 hematologic or non-hematologic events in the protocol window) [200]. Trial endpoints include proportion with ≥ 1 DLT, time-to-first DLT, dose reductions/delays, and relative dose intensity (RDI) [201]. Maintaining RDI ≥ 85% while reducing DLT burden is the pragmatic target; phenotype-guided prevention/personalization will be tested for its ability to lower DLTs without compromising RDI [202].

## Potential interventions are envisaged

### Pharmacologic: non-selective/selective β-blockers; central sympatholytics; ET-1–axis modulators

#### **β-blockers**

 Sustained β-adrenergic signaling fosters tumor growth, angiogenesis, metastasis, and immune suppression, providing a mechanistic rationale for β-blockade [100, 203, 204]. Propranolol down-regulates hypoxia programs in CRC models (↓HIF-1α/CAIX) [205] and has shown signal when added to standard care in angiosarcoma, albeit in small, non-randomized series [206]. Observational data in several tumors (e.g., epithelial ovarian cancer) suggest advantages for non-selective over β1-selective agents [207], but meta-analyses remain heterogeneous—benefit in BC/melanoma, neutral or inconsistent elsewhere—and are susceptible to confounding [208–210]. In bevacizumab-treated CRC, incidental β-blocker exposure correlated with improved PFS/OS [211]. Non-selective agents may better cover tumor-relevant β-axes, though receptor profiles differ across tumor and stromal compartments [208, 212, 213]. Immune remodeling with propranolol (CD4⁺ activation, monocyte shifts) offers additional biologic support for its use [214]. 

#### **Trial use**

Enroll patients with a high Sympathetic Overdrive Phenotype Score, implement a cardiology-supervised run-in, and apply prespecified cycle checkpoints (5-min HRV, HR/BP, ET-1; ±MSNA) to guide dose adaptation.

#### **Central sympatholytics**

Clonidine, methyldopa, guanfacine, and moxonidine reduce central sympathetic outflow [215–217]. Given the limited oncology evidence, reserve these agents for clearly high-score phenotypes with stringent BP monitoring and predefined stop rules; continue only with demonstrable HRV/ET-1 improvement [215].

#### **ET-1–axis modulators**

 ET-1 drives proliferation, invasion, EMT, angiogenesis, and chemoresistance [32, 218]. Endothelin-receptor antagonists enhance cytotoxic responses preclinically [219–222], but clinical results are mixed: macitentan plus temozolomide was tolerable in GBM without consistent biomarker change [223]; zibotentan did not improve OS in phase III CRPC despite earlier-phase signals [224–226]; data for bosentan remain sparse [227].

#### **Integration**

In ET-1–high phenotypes, test ERA add-ons using week-4 ET-1 change as the primary autonomic endpoint, HF/LF: HF as the key secondary, and perfusion/hypoxia MRI as exploratory; proceed only if autonomic targets are met without excess toxicity.

### Bio-behavioral health: sleep apnea treatment, structured exercise, stress modulation, vagal stimulation

#### **Sleep apnea treatment**

Obstructive sleep apnea (OSA) sustains sympathoexcitation through chemoreflex and baroreflex dysregulation [228, 229]. Continuous positive airway pressure (CPAP) consistently lowers MSNA and circulating catecholamines, although effects on global HRV are variable [230]. In oncology settings, CPAP adherence is associated with improved daytime symptoms in retrospective cohorts [231]; while large population studies show no clear reduction in cancer incidence, and meta-analytic data suggest only a trend [232, 233]. Notably, severe OSA and nocturnal hypoxia predict poorer survival and higher progression risk independent of stage and treatment [234]. Operationally, patients with a high autonomic–vascular score (HF below reference and/or ET-1 > ULN) should enter a CPAP or mandibular advancement device (MAD)–anchored bundle; de-escalation is triggered when ET-1 normalizes, and LF/HF remains below threshold for ≥ 1 cycle, whereas failure to meet targets prompts escalation.

#### **Structured exercise**

 Supervised training reduces MSNA in OSA [235] and, in cancer cohorts, improves HRV and sympathovagal balance — plausibly via convergent neuroimmune, endothelial, and oxidative-stress pathways [173, 236, 237].

#### **Stress modulation**

Chronic psychosocial stress and sleep loss activate the SNS/HPA axes and promote pro-tumor biology [238, 239]. The role of chronic stress in the etiology of cancer has been recently reviewed by Khan A et al. [240] and demonstrated experimentally by other researchers. In this regard, repeated restraint stress accelerated NMU-induced mammary carcinogenesis in rats - increasing incidence and multiplicity and shortening latency - linking sustained psychoemotional stress to tumor promotion [18]. Similarly, studies in mice exposed to unpredictable chronic stress showed a gastric tumor-promoting effect, which involved the participation of adrenergic receptors, hypoxia, and immune deregulation [241]. In oncology, CBT, MBSR, and HRV-biofeedback have been shown to increase vagal tone, reduce sympathetic predominance, improve mood and sleep, and modulate inflammatory/immune indices [130, 242–244]; early physiologic and symptom gains have been linked to better longer-term outcomes in selected settings [245]. Among survivors of breast and colorectal cancer, MBSR improves symptoms and cognition and alters inflammatory biomarkers, with benefits replicated across populations [246–250]; CBT and MBCT similarly reduce anxiety, depression, and fatigue, with CBT showing a modest advantage for fatigue in one trial [251]. Program monitoring should include HRV and ET-1 assessments at weeks 2 and 4; if predefined physiologic targets are not achieved, escalation to tVNS or addition of β-blockade is recommended.

#### **Vagal stimulation (tVNS/HRV-biofeedback)**

In patients who exhibit persistent vagal withdrawal, low HF power with elevated LF/HF, despite exercise and stress-modulation, we institute a structured autonomic rehabilitation program comprising HRV-biofeedback (15–20 min/day using resonance-frequency breathing) and/or auricular tVNS (20–30 min/day), delivered ≥ 5 days/week over one treatment cycle; sham control is recommended where feasible to permit mechanistic attribution [252]. The a priori response target at week 4 is restoration of HF into the local reference band with concurrent LF/HF reduction below the prespecified threshold; failure to meet targets prompts protocolized intensification or combination of modalities. Safety and objective response are tracked via weekly symptom checks and clinic assessments of HRV and ET-1 at baseline, week 2, and week 4, enabling data-driven adjustment and documentation of autonomic correction [253, 254].

## Conclusions, relevant syndromes, and 3PM-guided clinical implications in recommended steps

This manuscript positions chronic sympathoexcitation as a clinically actionable host phenotype with direct relevance to the malignant transformation, creation of pre-metastatic niches, tumor perfusion, hypoxia-linked resistance, and therapy tolerance. The proposed conceptual innovation is operational to move from the phenotype-specific stress chronification to individually measurable stage-specific biomarker-panels, health risk assessment, and predictive diagnostics, followed by individualized protection against health-to-disease transition in primary care and against disease progression in secondary care. The proposed 3PM-guided innovation is presented by the Graphical Abstract (see Fig. 1).

### Phenotyping is the recommended 1st step: screening and individualized protection against health-to-disease transition exemplified by the FSP carriers

The FSP is characterised by the stress chronification and sympathetic overdrive leading to the cyclic ischemia-reperfusion, particularly damaging for the small vessels and may cause a progressing small vessel disease, systemic low-grade inflammation, mitochondrial stress, and burnout. The latter is manifested in chronic fatigue and a spectrum of associated pathologies, including malignant transformations and premetastatic lesions. FSP is manifested early in life during pubertal maturation, which makes FS phenotyping instrumental for 3PM-guided screening and follow-up targeted prevention in primary healthcare. Indeed, a patient-friendly non-invasive approach utilising tear fluid multi-omics and mitochondria as vital biosensors is strongly recommended for the 3PM-guided healthcare applied to the FSP carriers [2].

Per scientific evidence, FSP/SOP carriers are predisposed to chronic dehydration, sterile inflammation, and related syndromes relevant to the malignant transformations. To this end, accumulated research data demonstrate a strong predisposition of the FSP carriers to chronic dehydration, Sicca syndrome (xerostomia, chronic hyposalivation, „dry mouth” syndrome), “burning mouth/tongue” syndrome, and vulvar-vaginal dryness [1, 255–257]. Further, a functional relationship between the FSP and Sjögren syndrome has been proposed [258]. Indeed, on one hand, a reduced or even suppressed thirst feeling characteristic of the FSP carriers frequently causes extensive body dehydration, resulting in systemic health risks. On the other hand, Sjögren syndrome (SS) is characterised by a progressive Sicca syndrome associated with symptoms and epigenetic dysregulation shared by both syndromes, such as altered sense regulation, vascular dysregulation, chronic sterile inflammation, and immune system deficits. These similarities are considered highly relevant for the malignant transformation risks in both syndromes [258].

Chronic pelvic pain syndrome and fibromyalgia often coexist and even overlap with other syndromes such as chronic fatigue syndrome, anxiety disorders, irritable bowel syndrome, interstitial cystitis, and chronic prostatitis. Chronification of stress is implicated in the pathogenesis of these syndromes and illnesses. The dominant sympathoexcitation, frequently accompanied by sterile inflammation, pain chronification, and mitochondrial stress or even mitochondrial burnout, is considered the key element of shared pathomechanisms [259–262]. In turn, mitochondrial stress and sterile inflammation are involved in malignant transformations [263].

Below, we illustrate FS phenotyping as a powerful tool of the 3PM-guided approach in primary care in order to protect affected individuals against health-to-disease transition.

#### "Dry mouth” syndrome – neglected risks in adolescents: A patient case analysis

A 20-year-old male, an excellent University student studying medicine; the strongly career-oriented person demonstrating by far the highest-than-average achievements. Anamnesis: since early childhood, the patient has suffered from acute and chronic otorhinolaryngologic infections: chronic tonsillitis is clinically manifested; strongly pronounced “dry mouth” syndrome with related discomfort of the oral cavity. Further examinations demonstrated high FSP scoring with particularly pronounced below-listed phenotype-specific symptoms and signs, namely:

- fully suppressed feeling of thirst and too low liquid intake over the day;
- strongly pronounced tendency towards perfectionism;
- frequently observed cold extremities even during the warm period, and feeling cold in the environment where the majority find the temperature rather comfortable;
- impaired wound healing;
- low BMI and blood pressure

amongst others, as reported earlier [1].

**Fig. 3:**
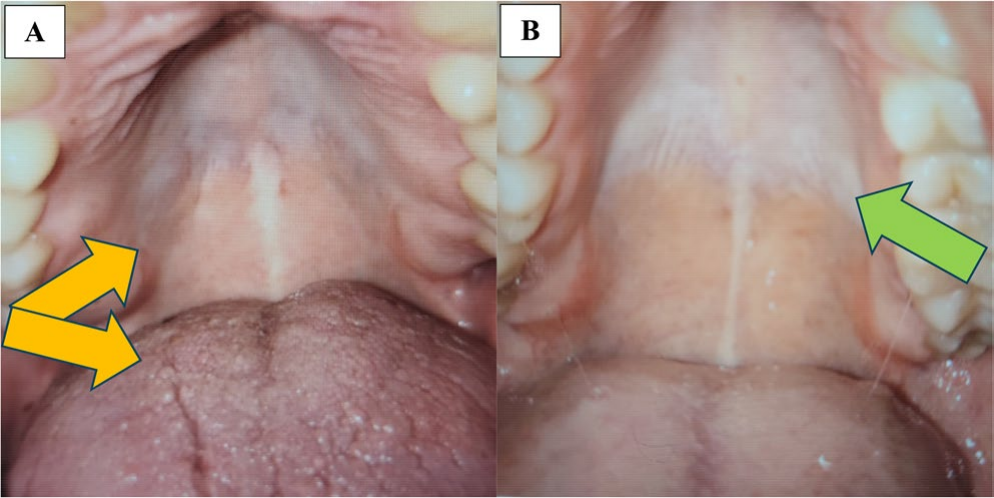
Diagnostics of xerostomia in dental practice: **A**. the image of a strongly hyposalivated (matt) roof of the mouth vs. a normally salivated roof of the mouth (**B**); the image is adapted from [1]

Figure 3 demonstrates diagnostics of the “dry mouth” syndrome (hyposalivation) in dental practice, frequently accompanied by the “burning mouth/tongue” syndrome.

The authors conclude that the “dry mouth” syndrome is more frequently diagnosed in individuals who are FSP carriers, compared with non-FSP individuals. Accumulated research data demonstrated that the “dry mouth” syndrome can be caused by acute and chronic stress exposure, metabolic impairments, and abnormal body weight, as well as Sjögren’s and Sicca syndromes, amongst others. The chronic hyposalivation, in turn, may indicate systemic dehydration and significantly contribute to several pathologies, including a strongly compromised protection of the oral cavity, chronic infections and inflammations, periodontitis, digestive disorders, and malignant transformations. Contextually, the “dry mouth” syndrome is considered a preventable health risk associated with the FSP in 3PM-guided primary care [1].

#### Vulvar-vaginal dryness: individualised patient profiles, risks and mitigating measures — FSP patient profile analysis

VD is considered a suboptimal health condition with preventable multi-factorial risk factors. However, if not adequately treated in both primary and secondary care, vulvar-vaginal dryness significantly increases the risk of genital female cancers.

The field-dedicated EPMA expert working group investigated the VD clinical manifestation in the FSP carriers against non-FSP individuals and concluded the phenotype characteristic features summarized below [256]:

*The most frequently pronounced FSP symptoms in premenopausal VD patients*

- perfectionistic personality;
- strongly reduced thirst perception but strongly pronounced smell perception;
- excessive vasoconstriction and feeling cold in the environment where the majority find the temperature rather comfortable;
- low BMI and blood pressure;
- frequent Headache, Dizziness, and Tinnitus.

*The most frequently pronounced FSP symptoms in postmenopausal VD patients*

- excessive vasoconstriction and feeling cold in the environment where the majority find the temperature rather comfortable;
- low blood pressure and low BMI in early adulthood;
- frequent dizziness, headache, and migraine with accompanying symptoms;
- prolonged sleep onset;
- chronification of pain.

Notably, about 20% of investigated VD patients reported on a delayed or even impaired wound healing, which they observed over a couple of years.

The authors emphasised the following strongly contributing risk factors:

A.  The well-known hormonal regulation changes characteristic for the peri/post-menopause, breastfeeding, intensive vaginal douching, cigarette smoking, cold medications, surgical removal of the ovaries, anti-oestrogen medication, anti-depressants, irradiation and chemotherapy, antihistamines, and premature ovarian insufficiency, amongst others.
B. Dehydration: adequate liquid intake is essential for physiologic vaginal lubrication; in contrast, whole-body dehydration causes Sicca syndrome and VD; to this end, due to significantly reduced thirst perception, FSP carriers are a priori predisposed to a reduced or even to the critically low liquid intake.
C. excessive vasoconstriction: significantly decreased peripheral blood flow may lead to a deficient vaginal lubrication and changes in vaginal innervation, collectively promoting vaginal dryness [256].
D. Psychological factors and chronification of stress overload: besides the above-explained collateral excessive vasoconstriction, a significant stress overload and anxiety negatively impact both libido and vaginal lubrication [256].

#### Clinically manifested malignant transformation: individualized protection of the FSP carriers against disease progression and rehabilitation of treated cancer patients in secondary care

##### FSP is relevant for pre-metastatic niches in BC – patient profile analysis.

The field-dedicated EPMA expert working group investigated FSP as a risk factor of the clinically manifested metastatic BC in pre- and post-menopausal women and concluded the subgroup-specific profiles summarized below [264].

*Shared FSP symptoms of pre- and post-menopausal metastatic BC patients*

- excessive vasoconstriction and feeling cold in the environment where the majority find the temperature rather comfortable;
- reduced thirst perception but strongly pronounced smell perception;
- frequent dizziness, headache, and migraine with accompanying symptoms.

*Particularly pronounced FSP symptoms in premenopausal metastatic BC patients*

- low blood pressure;
- tinnitus.

*Particularly pronounced FSP symptoms in postmenopausal metastatic BC patients*

- particularly reduced thirst perception;
- low BMI in early adulthood;
- strongly shifted circadian rhythms and prolonged sleep onset / reduced sleep duration and quality.

The authors proposed that the phenotype-specific epigenetic regulation predisposes FSP carriers to systemic hypoxic premetastatic niches, which may be created a long time before the breast malignancy is clinically manifested. Contextually, the FSP is instrumental for individualised patient profiles used for cost-effective personalised protection against health-to-disease transition in the area [264].

##### SOP as a functional modifier of the metastatic colorectal cancer progression and predictive power of mitochondrial fitness.

**Case 1**

**Fig. 4:**
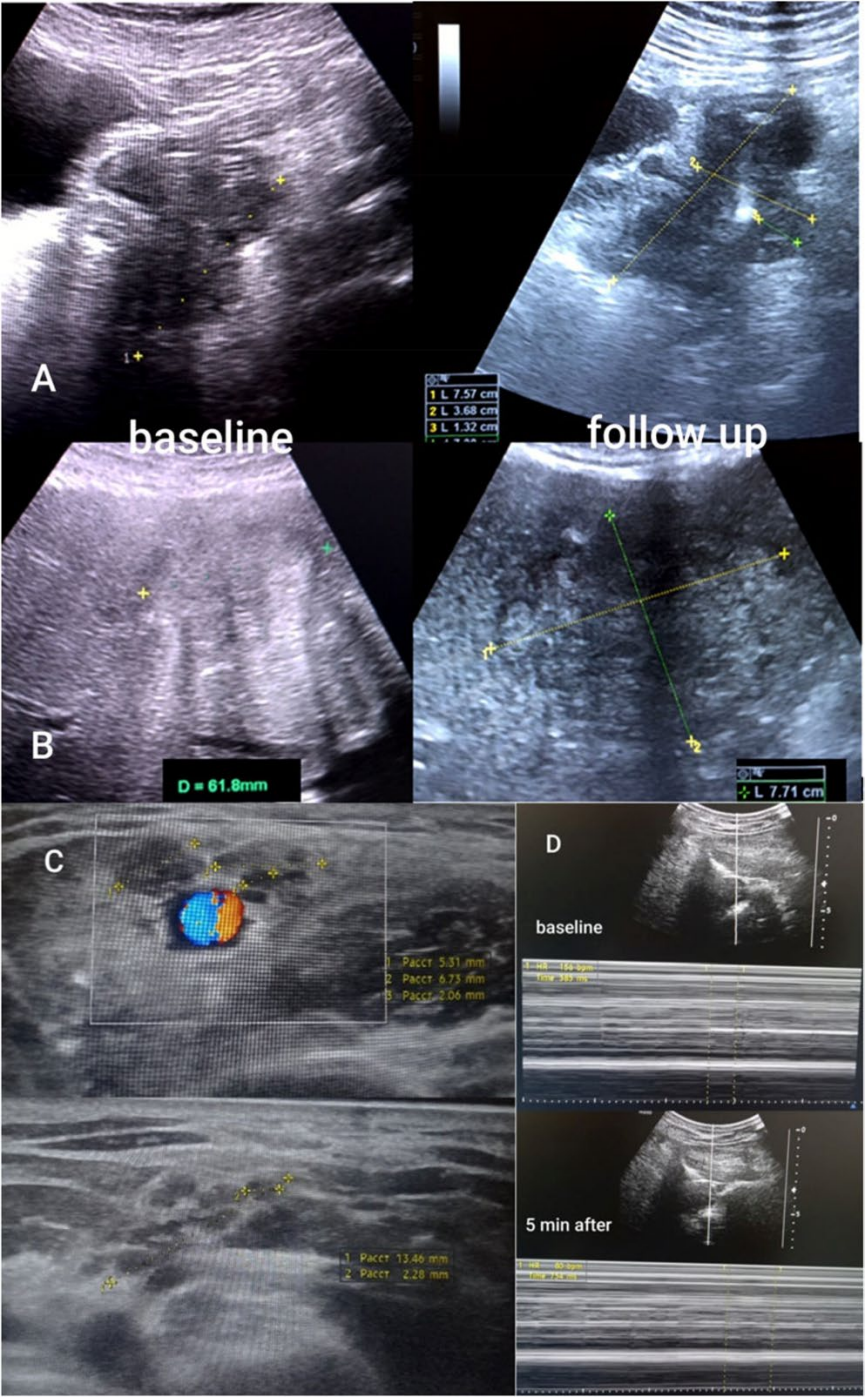
Multilevel ultrasound phenotyping of metastatic colorectal cancer of the patient with a strongly pronounced SOP; Left: baseline. Right: 6-month follow-up after chemotherapy. (**A**) Primary rectosigmoid tumor. Asymmetric wall thickening with preserved lumen patency despite morphologic progression, highlighting functional–structural dissociation; (**B**) Liver metastasis with fractal contour dynamics. Baseline lesion is relatively compact; follow-up shows contour irregularity, multi-scale segmentation, and satellite emergence. Fractal dimension increased from ≈ 1.08–1.14 to ≈ 1.28–1.38, reflecting rising structural heterogeneity due to combined progression and chemotherapy-induced architectural defragmentation; (**C**) Chemotherapy-induced axillary neuropathy. Altered nerve echotexture and enlargement consistent with oxaliplatin neurotoxicity; reversible after discontinuation; (**D**) Central sensitization marker and SOP modulation. Suboccipital/myodural imaging demonstrates abnormal oscillatory dynamics before intervention, normalizing immediately after ultrasound-guided dry needling. Oscillation frequency decreased from ~ 160/min to ~ 80/min (2-fold reduction), consistent with rapid modulation of sympathetic overdrive

A 65-year-old male with metastatic rectosigmoid carcinoma and liver metastases was observed and treated utilising multi-professional expertise and included tumor imaging, fractal morphometry, neuroautonomic assessment, and renal vascular profiling. While the primary tumor preserved partial lumen patency despite asymmetric wall thickening, hepatic metastases demonstrated progressive structural remodeling under chemotherapy. Contour-based fractal analysis revealed a marked increase in fractal dimension (FD ≈ 1.08–1.14 at baseline vs. ≈1.28–1.38 at 6 months), indicating rising spatial heterogeneity rather than simple volumetric growth. FD elevation reflected two concurrent processes: (i) clonal dispersion with satellite contour emergence (progression), and (ii) chemotherapy-induced architectural defragmentation. Thus, fractal dynamics captured biologically active disease under treatment pressure beyond size-based criteria [265]. These structural changes paralleled a clinically evident Sympathetic Overdrive Phenotype characterized by stress intolerance, autonomic reactivity, and pain amplification. Severe opioid-refractory shoulder pain resolved rapidly after ultrasound-guided dry needling, supporting dominant central sensitization. Suboccipital imaging demonstrated abnormal oscillatory spinal-root/myodural dynamics that normalized immediately after intervention, with oscillation frequency decreasing from ~ 160/min to ~ 80/min (2-fold reduction), consistent with acute modulation of sympathetic overdrive. Oxaliplatin-induced neuropathy was visualized by nerve ultrasound and resolved after drug discontinuation. Renal Doppler revealed elevated intrarenal resistive index in the context of hypertension, indicating increased sympathetic vascular tone and influencing chemotherapy tolerance and contrast-imaging decisions. This patient case illustrates SOP as a systemic functional accelerator influencing tumor heterogeneity, pain processing, vascular regulation, and therapeutic response. Fractal ultrasound biomarkers served as integrative indicators of tumor–neuroautonomic interaction within the paradigm of a proactive medical approach (Fig. 4).

**Case 2**

Three years ago, a 52-year-old male patient was diagnosed with colorectal cancer and metastases to the stomach, liver, and lung, and successfully treated by surgery and chemotherapy. The patient cares about healthy lifestyle habits, high-quality meals, and sleep. Nevertheless, the patient demonstrates a strongly pronounced SOP: meticulous personality and cold extremities are evident. Due to persistent chronic fatigue, the patient underwent a tear fluid test to analyze mitochondrial fitness, followed by a multi-professional consultation [266]. The tear fluid test demonstrated mitochondrial burnout as a pre-stage of the mitochondrial burnout. Four months later, a recurrence of the colorectal carcinoma was diagnosed at an early stage and successfully treated. The patient is considered cured now: CT, CEA, CA 19 − 9, and CA 72 − 4 are all regularly monitored and remain negative. Currently, the patient undergoes an individualized rehabilitation program including mitochondrial fitness support under the supervision of a multi-professional group of experts [6].

**Case 3**

A 53-year-old female patient was diagnosed with colorectal cancer. The patient is a carrier of highly scored FSP. The most pronounced symptoms recorded are the following ones:

- perfectionism in almost everything (the patient made an excellent academic career);
- low BMI = 16;
- low blood pressure;
- reduced sleep duration and quality characterized by nocturnal breaks and difficulty falling asleep;
- highly pronounced stress (including cold stress) sensitivity;
- vaginal dryness and associated sexual dysfunction.

The patient reports on an adequate physical activity and high-quality dietary habits.

The patient reports on anxiety: psychometric evaluations indicate moderate depression (PHQ-9 score: 10) requiring regular psychological consultations. Further, the patient reported on a strongly pronounced chronic fatigue observed a long time before the diagnosis.

Five weeks after the colorectal cancer surgery, the responsible medical center requested the tear fluid test to evaluate mitochondrial fitness and to design personalized rehabilitation algorithms. The test demonstrated a strongly pronounced mitochondrial burnout.

##### Mitochondria‑based holistic 3PM approach for individualized rehabilitation of treated cancer patients.

Mitochondria-based approach is considered effective and was strongly recommended for advanced diagnostics and treatments applied to the FSP carriers in their protection against cancer [257, 267, 268].

Regarding the innovative diagnostic technologies, tear fluid is considered an optimal source of information and is strongly recommended for the FSP carriers´ screening early in life [269]. By operating via mitochondrial function, the proposed rehabilitation approach triggers systemic effects holistically, positively affecting energy metabolism, repairing mechanisms, physical shape, and mental health. Highly effective rehabilitation algorithms are always tailored to individualized patient profiles: Mitochondrial Biosensorics Check-Up is crucial for physical fitness and exercise intervention quality [266]. The proposed innovation integrates mitochondrial health assessments utilizing mitochondrial homeostasis biomarkers in tear fluid as a non-invasive diagnostic tool, tailored nutraceuticals, and lifestyle adjustments [6, 270].

### 3PM application of mitochondrial biosensorics is the recommended 2nd step

Mitochondrial stress, according to evidence, is attributable to the chronic ischemia-reperfusion, which is one of the key pathomechanisms linked to the SOP / FSP with complications. Besides the systemic energy supply deficits, chronification of the mitochondrial stress results in compromised mitochondrial homeostasis. Consequently, changes in mitochondrial homeostasis are an attractive target for individualised health risk assessment and treatments tailored to individualised patient profiles in primary and secondary care, including individualised pre- and rehabilitation programmes [2, 6, 266]. To this end, mitochondrial medicine is considered highly relevant for diagnostics and treatments of FSP carriers [263].

### Follow-up step 3: Treatments tailored to the individualized patient profile

Personalized treatment algorithms in primary (protection against health-to-disease transition) and secondary (protection against disease progression) care and effective rehabilitation programs are recommended to follow individualized patient profiles. To improve individual outcomes, specifically in the subpopulation of FSP/SOP carriers, the following key components should be considered:

- psychosomatic patterns;
- increased stress sensitivity;
- systemic mitochondrial stress and reduced energy supply;
- systemic ischemia-reperfusion damage;
- systemic sterile inflammation;
- chronification of pain;
- altered drug sensitivity;
- compromised quality of sleep.

More explanatory details are provided in the recently published article “How to use an extensive Flammer syndrome phenotyping for a holistic protection against health-to-disease transition - facts and practical recommendations“ [2]. 

## Data Availability

No datasets were generated or analysed during the current study.

## Abbreviations

3PM: Predictive, preventive, and personalized medicine
BOLD (MRI): Blood-oxygen-level–dependent magnetic resonance imaging
BP: Blood pressure
BRS: Baroreflex sensitivity
BC: Breast cancer
CAIX: Carbonic anhydrase IX
CBT: Cognitive-behavioural therapy
CD39: Ectonucleoside triphosphate diphosphohydrolase-1 (ENPP1)
CD73: 5′-Nucleotidase ecto (NT5E)
CRPC: Castration-resistant prostate cancer
CRC: Colorectal cancer
CPAP: Continuous positive airway pressure
CTCAE: Common Terminology Criteria for Adverse Events
CV: Coefficient of variation
CXCL12/CXCR4 axis: chemokine ligand/receptor pair (SDF-1/CXCR4)
DCE-MRI: Dynamic contrast-enhanced magnetic resonance imaging
DLT: Dose-limiting toxicity
ECM: Extracellular matrix
EMT: Epithelial–mesenchymal transition
ERA: Endothelin-receptor antagonist
ETA: Endothelin A
ET-1: Endothelin-1
FACIT-F: Functional Assessment of Chronic Illness Therapy – Fatigue subscale
fMRI: Functional magnetic resonance imaging
FMD: Flow-mediated dilation
FS: Flammer Syndrome
FSP: Flammer Syndrome Phenotype
GBM: Glioblastoma
GLUT1: Glucose transporter 1
HF: High-frequency power (parasympathetic/vagal HRV band)
HF-HRV: High-frequency heart-rate variability
HIF: Hypoxia-inducible factor
HR: Heart rate
HRV: Heart-rate variability
HPA axis: Hypothalamic–pituitary–adrenal axis
HPV: Human papillomavirus
HV: Hypoxic volume (MRI-derived)
LDHA: Lactate dehydrogenase A
LF: Low-frequency power (mixed autonomic HRV band)
LF/HF: Ratio of low- to high-frequency HRV power (sympathovagal balance)
MAD: Mandibular advancement device
MBCT: Mindfulness-based cognitive therapy
MBSR: Mindfulness-based stress reduction
MMPs: Matrix metalloproteinases
MSNA: Muscle sympathetic nerve activity
NF-kB: Nuclear factor kappa-B
NMU: Neuromedin U
OE-MRI: Oxygen-enhanced magnetic resonance imaging
OS: Overall survival
OSA: Obstructive sleep apnoea
PFS: Progression-free survival
RDI: Relative dose intensity
ROS: Reactive oxygen species
SDNN: Standard deviation of normal-to-normal intervals
SOP: Sympathetic overdrive phenotype
SDF-1: Stromal cell–derived factor 1 (CXCL12)
SNS: Sympathetic nervous system
tVNS: Transcutaneous vagus nerve stimulation
TME: Tumor microenvironment
TOLD (MRI): Tissue-oxygen-level–dependent magnetic resonance imaging
ULN: Upper limit of normal (assay reference)
VD: Vaginal dryness
VEGF: Vascular endothelial growth factor
